# Efficacy and safety of pomalidomide, bortezomib, and dexamethasone combination chemotherapy for newly diagnosed multiple myeloma: POMACE Phase II Study

**DOI:** 10.1038/s41408-023-00816-8

**Published:** 2023-03-24

**Authors:** Fen Saj, Yadav Nisha, Prasanth Ganesan, Smita Kayal, Rakhee Kar, Dhanapathi Halanaik, Biswajit Dubashi

**Affiliations:** 1 Department of Medical Oncology, Jawaharlal Institute of Postgraduate Medical Education and Research (JIPMER), Puducherry, India; 2 Department of Pathology, Puducherry, India; 3 Department of Nuclear Medicine, Puducherry, India

**Keywords:** Chemotherapy, Chemotherapy

## Abstract

Bortezomib, lenalidomide, and dexamethasone induction chemotherapy (VRd), followed by autologous stem cell transplantation (ASCT), are the standard of care for patients with newly diagnosed multiple myeloma (NDMM). Pomalidomide is currently approved for relapsed-refractory multiple myeloma. This single-arm, open-label, phase 2 study was the prospective evaluation of the efficacy and safety of bortezomib, pomalidomide, and dexamethasone (VPd) induction for NDMM. We used Fleming’s two-stage design for sample size calculation. We included transplant-eligible and ineligible patients aged 18–75 years in the study. The patients received four cycles of VPd induction followed by response assessment. Thirty-four patients were included in the study, of which 31 completed all four cycles of induction. The median age was 52 years (32–72). Thirty (91%) patients had multiple myeloma, and three had multiple plasmacytomas with less than 10% bone marrow involvement. Nine (27%) had ISS-I, 9 (27%) had ISS-II, and 15 (46%) had ISS-III myeloma. Three patients had high-risk cytogenetic abnormalities. After four cycles of VPd induction, ten patients (32%) achieved stringent CR, nine had CR (29%), eight (26%) had VGPR, and 4 (13%) had PR. Fifteen (48%) had a complete metabolic response (CMR) on PET-CT. Two patients developed SAEs. Anemia was the most common hematological toxicity. Peripheral neuropathy and constipation were the most common non-hematological toxicities. Patients with ≥VGPR had significantly better 12-month PFS than those with PR. Patients with ≥VGPR and CMR on PET-CT had significantly better 12-month OS. Our study showed VPd induction is safe and efficacious in NDMM. Further Phase 3 studies are necessary to establish the superiority and survival benefits.

## Introduction

The recommended standard of care in newly diagnosed multiple myeloma (NDMM) patients is induction chemotherapy, consolidation with autologous stem cell transplantation (ASCT) with high-dose melphalan conditioning if transplant-eligible, followed by maintenance [1]. Multiple myeloma remains a disease where curability is difficult [2]. The goal of therapy in transplant-eligible and ineligible patients is to maximize the depth of tumor reduction, which correlates with improved progression-free survival (PFS) and overall survival (OS) [3]. A combination of bortezomib with lenalidomide and dexamethasone (VRd) is one of the frontline treatment options for NDMM [4]. In the PETHEMA/GEM2012 study, the rates of complete response (CR) and very good partial response or better (≥VGPR) after four cycles of induction with the VRD regimen were 33.4% and 63.8%, respectively [5].

Drugs initially approved for relapsed and/or refractory MM (RRMM), like daratumumab and carfilzomib, have been introduced for the frontline treatment of NDMM to achieve higher rates of response [6, 7]. Pomalidomide is a third-generation immunomodulatory drug currently approved for RRMM [8]. Pomalidomide exerts potent immune-enhancing activity by binding to the target protein, cereblon [9]. Compared to lenalidomide, pomalidomide demonstrates higher potency against cereblon, and has a unique gene activation profile and substrate degradation kinetics [10]. It has shown activity in lenalidomide-resistant cell lines and animal models [11, 12].

The data on the use of pomalidomide in NDMM is limited. We undertook the POMACE study, an open-label, single-arm, phase 2 study to evaluate the efficacy and safety of pomalidomide in NDMM.

## Methods

Our study was a single-center, single-arm, open-label, phase 2 clinical trial with enrolment from July 2020 to December 2021. The trial was approved by the institutional ethics committee (JIP/IEC/2020/059) and the Clinical Trials Registry of India (CTRI/2020/09/027547). It was conducted in accordance with the Declaration of Helsinki and the Indian Council of Medical Research (ICMR) Guidelines for Good Clinical Practice. All patients provided written informed consent.

### Patients

Treatment naïve, NDMM, aged between 18–75 years, and who had an Eastern Cooperative Group Performance Status (ECOG PS) 2 or below were eligible. Previous local radiotherapy should have been completed at least two weeks before the study inclusion. Patients were excluded if they had ≥grade 2 peripheral neuropathy, absolute neutrophil count (ANC) < 1000, platelet count <50,000, total bilirubin and transaminases elevated three or more times the upper limit of normal, creatinine clearance <15 ml/min, or other significant and active comorbidities or infections. Patients with plasma cell leukemia, amyloidosis, and POEMS syndrome were excluded.

### Intervention

Four courses of induction chemotherapy (each cycle of 4 weeks) of subcutaneous bortezomib 1.3 mg/m^2^/day on days 1, 8, 15, and 22 (capped at 2 mg due to financial considerations); oral pomalidomide on days 1 to 21 (2 mg/day dose in cycle 1 and 4 mg/day dose from cycle two onwards); and oral dexamethasone 40 mg/day on days 1, 8, 15, 22 were given (VPd regimen). Transplant-eligible patients who achieved at least VGPR or better could proceed to ASCT after a minimum of four cycles. VPd was given for a maximum of six cycles, followed by bortezomib and dexamethasone (Vd) till ASCT. Stem cell mobilization was done with granulocyte colony-stimulating factor (GCSF) with or without plerixafor. The conditioning regimen used was high-dose melphalan (140–200 mg/m^2^). After ASCT, the patients continued with maintenance. Transplant-ineligible patients continued the three-drug induction for nine cycles, followed by maintenance. The patients in the Revised International staging system (R-ISS) stage I received pomalidomide maintenance, while stages II and III received bortezomib maintenance. All patients received unless contraindicated, aspirin prophylaxis (75 mg daily), antiviral therapy (acyclovir 400 mg twice daily) for prevention of herpes zoster, and bisphosphonates (zoledronic acid).

### Assessments

The primary endpoint was to determine the rates of very good partial response or better (≥VGPR) after four courses of VPd. The secondary endpoints were the assessment of safety, PFS, and OS.

At baseline, all patients underwent serum protein electrophoresis (SPEP) with quantification of M-protein, immunofixation (IF), serum free light chain assay (SFLC), estimation of beta two microglobulin, bone marrow aspiration-biopsy with testing for cytogenetic abnormalities using fluorescent in-situ hybridization (FISH), and PET-CT scanning as skeletal imaging. The complete blood count, renal and liver function tests, plasma glucose, and serum calcium levels were estimated at baseline and before each cycle. Quantifying M-protein in blood was done at the end of two cycles as an interim assessment for patients with a baseline elevation. Complete response assessments, including SPEP, IF, SFLC, bone marrow biopsy, and PET-CT scanning were done at the end of four cycles. The response was assessed according to the International Myeloma Working Group (IMWG) Uniform Response Criteria [13]. Toxicity and tolerance were evaluated at the end of each cycle and during follow-up. Adverse events (AEs) were graded using the National Cancer Institute Common Terminology Criteria version 5.0. The Data cutoff for analysis was in July 2022.

### Sample size calculation and Statistics

Fleming’s two-stage design was used for sample size calculation in this study. The number of patients achieving ≥ VGPR using the VRD regimen was approximately 65% [5]. We estimated that VPd induction would produce a ≥ VGPR rate of 80%. In the first stage, 31 patients were accrued. If there were 21 or fewer responses in these 31 patients, the study would have been stopped for futility. If there were 26 or more responses in 31 patients, the study would have been stopped, and the null hypothesis rejected. Otherwise, 28 additional patients would have been accrued for a total of 59. The null hypothesis would have been rejected if 45 or more responses were observed in 59 patients. This design would yield a type I error rate of 0.05 and a power of 0.8 when the true response rate was 80%. Response rates and toxicities were calculated as percentages. Survival results were analyzed using Kaplan-Meir curves. Analysis was performed using SPSS software version 21.

## Results

### Patients

**Fig. 1:**
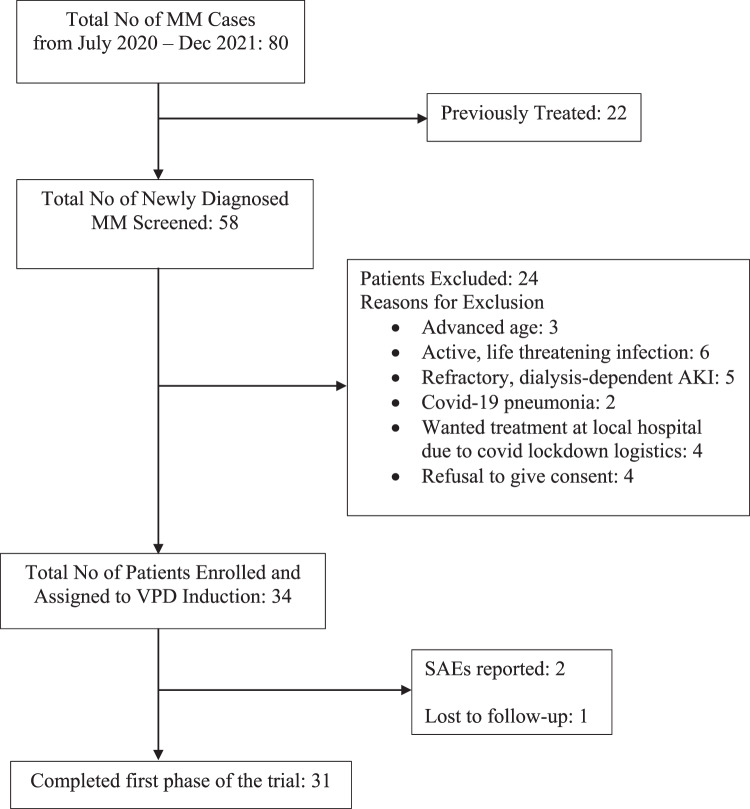
Consort of the study population.

During the study period, eighty multiple myeloma patients had registered, of which 58 were NDMM. Thirty-four patients were found to be eligible (Fig. 1). Thirty-one patients who completed at least four cycles of induction were included in the analysis for response rates. The median follow-up duration was 14 months.

The median age was 52 years (32–72), and 17 (55%) were males. Twenty patients (61%) had ECOG performance status 0–1. Thirty patients (91%) had multiple myeloma, and three had multiple skeletal plasmacytomas with bone marrow involvement <10%. The International Staging System (ISS) Stage was I in 9 (27%), II in 9 (27%), and III in 15 (46%) patients. High-risk cytogenetic abnormalities (defined as the presence of Del 17p13, t (4;14), t(14;16) were present in three (9%) patients. High LDH (normal range: 60–200 IU/L, high as defined by value >200 IU/L) was present in nine (31%) patients. Baseline lab and disease characteristics are summarized in Tables 1, 2, respectively.

**Table 1:** Laboratory characteristics of the study patients (*N* = 33).

Laboratory characteristic (*n*)	
Median Hemoglobin (g/dl, *n* = 33)	9.9 (4.8–16.3)
Median Total WBC count (cells/μL, *n* = 33)	7600 (3610–14,350)
Median Platelet count (cells/μL, *n* = 33)	258,000 (10,300–460,000)
Median LDH (IU/L, *n* = 29)	188 (94–516)
Patients with elevated LDH (defined as >200IU/L, *n* = 29)	9 (31)
Median Creatinine (mg/dl, *n* = 33)	0.8 (0.3–8.7)
Median Calcium (mg/dl, *n* = 33)	9.7 (8–14.4)
Median Albumin (mg/dl, *n* = 33)	3.6 (1.8–5.1)
Median beta 2 microglobulin (mg/dl, *n* = 33)	4.2 (1.6–20)

**Table 2:** Disease characteristics of the study patients (*N* = 33).

**Diagnosis (*n*** = 33)	
**CRAB Features (*n*** = **33)**	
Anemia (Hb <10 g/L)	17 (52)
Renal Dysfunction (Creatinine >2 mg/dl)	9 (27)
Hypercalcemia (corrected Ca >11 mg/dl)	10 (30)
Bone lesions (on imaging)	31 (94)
**Immunofixation (*n*** = **33)**	
IgG (*n* = 22, 67)	Kappa—17 (52)
	Lambda—5 (15)
IgA (*n* = 3, 9)	Kappa—2 (6)
	Lambda—1 (3)
Light chain disease (*n* = 6, 18)	Kappa—3 (9)
	Lambda—3 (9)
Negative (*n* = 2, 6)	2 (6)
**Skeletal imaging (*n*** = **33)**	
PET-CT	32 (97)
MRI/CT	0
Skeletal survey (X-ray)	1 (3)
**Skeletal involvement (*n*** = **33)**	
Axial only	4 (12)
Appendicular only	0
Both	27 (82)
Not applicable	2 (6)
**FISH testing (*n*** = **33)**	
Yes	28 (85)
No	5^a^
**FISH report (*n*** = **28)**	
No high-risk cytogenetic abnormalities	17 (61)
Del 17p13	2 (6)
Del 13q14	7 (25)
t(4,14)	0
t(11,14)	1 (4)
t(14,16)	1 (4)
**ISS stage (*n*** = **33)**	
I	9 (27)
II	9 (27)
III	15 (46)
**R-ISS stage (*n*** = **33)**	
I	6
II	12
III	10
Not applicable	5^b^

^a^(three patients-minimal BM involvement, two-financial constraints).

^b^(FISH analysis not done in 3 patients due to bone marrow involvement <10% and in 2 patients due to financial constraints).

### Efficacy

The overall response rate was 100%. Ten patients (32%) achieved stringent CR, nine had CR (29%), eight (26%) had VGPR, and 4 (13%) had PR. Twenty-seven (87%) patients had ≥VGPR, the study’s primary endpoint. Details of response rates are summarized in Table 3.

**Table 3:** Response assessment parameters of the study patients.

**Response as per IMWG Criteria at the end of 4 VPD Cycles (*n*** = 31)	
sCR	10 (32)
CR	9 (29)
VGPR	8 (26)
PR	4 (13)
SD	0
PD	0
≥VGPR	27 (87)
**PET-CT at the end of 4 VPD cycles (*n*** = 31)	
Metabolic complete response	15 (48)
Partial response	16 (52)
Progressive disease	0

At a median follow-up of 14 months (range: 8–21 months), nine patients had died, and five events of disease progression happened. PFS and OS at 12 months were 67.7% and 70.7%, respectively, in the intent-to-treat population. PFS at 12 months with or without ASCT in patients who had ≥VGPR (*n* = 27) was 87.1% compared to those who had PR (*n* = 4) was 50% (*p* = 0.03). OS at 12 months with or without ASCT in patients with ≥VGPR was 90.3% compared to those with PR at 50% (*p* = 0.002). PFS at 12 months in those patients with a complete metabolic response (CMR) in PET/CT done after four cycles (*n* = 15, 48%) was 93.3% as compared to those who had a partial response was 42.4% (*n* = 16, 52%) (*p* = 0.06). OS at 12 months in patients with CMR in PET/CT after four cycles were 100% compared to those with a partial response of 40.6% (*p* = 0.03).

### Safety

The most common (≥10%) hematological adverse events (AEs) of any grade were neutropenia (*n* = 8, 24%), anemia (*n* = 7, 21%), and thrombocytopenia (*n* = 5, 15%). Grade 3 anemia requiring blood transfusion was noted in three patients (9%). Peripheral sensory neuropathy (*n* = 9, 27%), fatigue (*n* = 9, 27%), and constipation (*n* = 8, 24%) were the most common (≥ 10%) non-hematological adverse effects of any grade. Two patients (6%) developed grade 3 peripheral sensory neuropathy requiring dose modification of bortezomib. One patient required a 50% dose reduction of pomalidomide (maintained at 2 mg for all cycles) due to pre-existing chronic kidney disease. Non-neutropenic infections of any grade happened in three (9%) patients, two of which were upper respiratory tract infections. Two patients developed serious AEs resulting in death. Of these two patients who died, one died of febrile neutropenia, cellulitis, and septic shock, while the other died of complications of non-neutropenic acute gastroenteritis. During follow-up, seven patients died, and five developed disease progression. Three patients died of covid pneumonia, one died of a road traffic accident, and one due to complications of myocardial infarction, all beyond the study period. Two patients who developed disease progression while on follow-up died of infection. Out of the five patients who developed progressive disease, all patients have been screened for salvage second-line chemotherapy, including carfilzomib and/or daratumumab-based regimens. Three patients were found unfit, and two are currently on second-line treatment. The toxicities, including grade 3/4 toxicities, are shown in Table 4.

**Table 4:** Adverse events of the study patients (*n* = 33).

	Any Grade	Grade 3, 4	Grade 5
Hematological toxicities			
Anemia	7 (21)	3 (9)	
Neutropenia	8 (24)	1 (3)	
Febrile neutropenia	1 (3)	0	1 (3)
Thrombocytopenia	5 (15)	0	
Non-hematological toxicities			
Mucositis	0	0	
CINV	2 (6)	0	
Diarrhea	0	0	
Constipation	8 (24)	0	
Peripheral neuropathy	9 (27)	2 (6)	
Fatigue	9 (27)	0	
Myalgia	2 (6)	0	
Venous thromboembolism	0	0	
Sedation	3 (9)	0	
Infection, non-neutropenic	1 (3)	0	1 (3)

### Stem cell harvest and engraftment

Of the 31 patients, 27 (87%) were transplant-eligible based on age ≤70 years and fitness status. Eleven (35%) proceeded to ASCT. The median duration from the start of induction to transplant was 8.5 months (5.4–12mos). The median number of cycles before ASCT was 8 (5–11), of which six were VPd, and the rest were Vd. GCSF was used in all patients, and plerixafor was used in six (55%) patients. A second session of apheresis was not required in any of the patients. Median CD34^+^ cell yield was 6.3 × 10^6^ cells/kg (2.8 × 10^6^–21.1 × 10^6^). The median duration of neutrophil engraftment was nine (8–11), and platelet engraftment was eleven (10–12) days, respectively.

## Discussion

One of the main goals of induction chemotherapy in newly diagnosed MM is achieving the deepest possible response, which may influence long-term outcomes [14]. This phase 2 study, a prospective investigation of the bortezomib-pomalidomide-dexamethasone combination in newly diagnosed MM, yielded a 100% response rate. After four cycles of VPd, 87% of patients achieved VGPR or better (primary endpoint), and 61% were CR or better. On PET-CT, 48% had achieved a complete metabolic response. These high response rates were similar to those achieved with carfilzomib and daratumumab in NDMM [6, 7, 15]. Another phase 2 study of VPd induction in NDMM reported similarly high response rates, sCR of 24%, and VGPR of 76% [16].

In our study, 48% of patients achieved complete metabolic response (CMR) on PET/CT. 18F-FDG-PET/CT is comparable to multiparameter flow cytometry to define minimal residual disease (MRD) in MM [17]. MRD-negative status is a strong prognostic indicator for PFS and OS, surpassing CR achievement [18]. Complete metabolic response on PET/CT correlates with PFS and OS [19]. Our study observed that patients with CMR on PET/CT had significantly better overall survival at one year than those with partial response. We welcome more studies with longer follow-ups to establish the role of PET-CT as a standard imaging modality after the completion of induction chemotherapy in MM.

The VPd regimen showed a manageable toxicity profile in our study. Two early deaths happened in the first cycle, of which one could be directly attributed to the drug. The first patient, a long-standing diabetic, died of febrile neutropenia and septic shock secondary to bilateral lower limb cellulitis. The second patient presented with a pre-renal form of acute kidney injury following an episode of community-acquired acute gastroenteritis and was non-neutropenic at presentation. Only one patient required dose modification for pomalidomide because of chronic kidney disease. None of the patients discontinued therapy. VPd was safe and effective in patients with advanced age and comorbidities. The OPTIMISSM study, which used a VPd combination for RRMM, showed anemia of any grade in 27% of patients and grade 3/4 in 13% of patients, respectively [8]. Similar rates were observed in our study. However, the rates of any grade of neutropenia and thrombocytopenia were 46 and 37%, respectively, in the OPTIMISSM study. Both these rates are higher than our study, probably because the former studied relapsed-refractory patients. Also, in the former study, pomalidomide was delivered at a dose of 4 mg/day from day 1–14, and the cycles were repeated every 21 days. Hematological toxicities due to chemotherapy are reportedly more common in the first cycle [20]. Compared to the former study, we delivered a 50% dose (2 mg) in the first cycle and 4 mg from cycle two onwards. This fact may also explain our study’s lower rates of hematologic AEs. Another reason for the higher rates of thrombocytopenia (37%) and peripheral sensory neuropathy (48%) of any grade in the former study could be because they used bortezomib at a dose of 1.3 mg/m^2^, while we capped the dose at 2 mg as part of the institutional protocol. Bortezomib-induced thrombocytopenia and peripheral sensory neuropathy are reported to be dose-dependent [21, 22]. Capping the dose at 2 mg may explain the lower incidence of these AEs. Though rare, atrial fibrillation, cardiac failure, and myocardial infarction have been reported with pomalidomide [23]. One of the patients who expired while on follow-up had a myocardial infarction. Even though he had other risk factors like diabetes, hypertension, renal dysfunction, and smoking history, we are unsure if pomalidomide also contributed to the risk for an acute cardiac event. Whether renal dysfunction makes the patients more prone to adverse events needs to be explored.

The median duration from diagnosis of multiple myeloma to ASCT is eight months. This duration is earlier than other studies from the country where the median duration was 10–10.5 months [24]. The primary reasons for less percentage of patients undergoing transplants were financial constraints and poor social support, as reported by other studies from the developing world [25]. Pomalidomide did not interfere with stem cell mobilization, and all the patients who proceeded to ASCT could complete the harvest with or without plerixafor support. None of the patients required a second harvest procedure. The median duration of neutrophil and platelet engraftment was comparable to patients treated with other regimens [26].

The PFS and OS at one year in our study were 67.7% and 70.7%, respectively. These rates are significantly lesser than the reported survival rates of myeloma in the modern era [27]. One of the reasons could be a short follow-up duration. Two deaths were reported as SAEs, and four deaths beyond the trial period were due to reasons not directly attributable to the study drug. This may have lowered the survival statistics. Hence, studies with larger sample sizes and longer follow-ups are necessary for data on real survival outcomes.

A few limitations of our study were that FISH for cytogenetic abnormalities could not be carried out in all the patients. Due to logistical difficulties, the assessment of MRD using multiparameter flow cytometry couldn’t be performed.

We conclude that induction chemotherapy using bortezomib, pomalidomide, and dexamethasone is safe and efficacious in newly diagnosed multiple myeloma. This regimen achieved higher rates of very good partial response and better than the conventionally used bortezomib-lenalidomide-dexamethasone regimen. Further Phase 3 studies with longer follow-ups are necessary to establish the superiority and survival benefit of the pomalidomide-based regimen for myeloma induction.

## Supplementary information

Reproducibility Checklist

## Data Availability

Data will be available on reasonable request.

## References

[1] Mikhael J, Ismaila N, Cheung MC, Costello C, Dhodapkar MV, Kumar S (2019). Treatment of Multiple Myeloma: ASCO and CCO Joint Clinical Practice Guideline. J Clin Oncol..

[2] Ravi P, Kumar SK, Cerhan JR, Maurer MJ, Dingli D, Ansell SM (2018). Defining cure in multiple myeloma: a comparative study of outcomes of young individuals with myeloma and curable hematologic malignancies. Blood Cancer J.

[3] van de Velde HJK, Liu X, Chen G, Cakana A, Deraedt W, Bayssas M (2007). Complete response correlates with long-term survival and progression-free survival in high-dose therapy in multiple myeloma. Haematologica.

[4] Callander NS, Baljevic M, Adekola K, Anderson LD, Campagnaro E, Castillo JJ (2022). NCCN Guidelines® Insights: Multiple Myeloma, Version 3.2022. J Natl Compr Cancer Netw.

[5] Rosiñol L, Oriol A, Rios R, Sureda A, Blanchard MJ, Hernández MT (2019). Bortezomib, lenalidomide, and dexamethasone as induction therapy prior to autologous transplant in multiple myeloma. Blood.

[6] Voorhees PM, Kaufman JL, Laubach J, Sborov DW, Reeves B, Rodriguez C (2020). Daratumumab, lenalidomide, bortezomib, and dexamethasone for transplant-eligible newly diagnosed multiple myeloma: the GRIFFIN trial. Blood.

[7] Jasielec JK, Kubicki T, Raje N, Vij R, Reece D, Berdeja J (2020). Carfilzomib, lenalidomide, and dexamethasone plus transplant in newly diagnosed multiple myeloma. Blood.

[8] Richardson PG, Oriol A, Beksac M, Liberati AM, Galli M, Schjesvold F (2019). Pomalidomide, bortezomib, and dexamethasone for patients with relapsed or refractory multiple myeloma previously treated with lenalidomide (OPTIMISMM): a randomised, open-label, phase 3 trial. Lancet Oncol.

[9] Holstein SA, Hillengass J, McCarthy PL (2018). Next-Generation Drugs Targeting the Cereblon Ubiquitin Ligase. J Clin Oncol.

[10] Lopez-Girona A, Mendy D, Ito T, Miller K, Gandhi AK, Kang J (2012). Cereblon is a direct protein target for immunomodulatory and antiproliferative activities of lenalidomide and pomalidomide. Leukemia.

[11] Rychak E, Mendy D, Shi T, Ning Y, Leisten J, Lu L (2016). Pomalidomide in combination with dexamethasone results in synergistic anti-tumour responses in pre-clinical models of lenalidomide-resistant multiple myeloma. Br J Haematol..

[12] Ocio EM, Fernández-Lázaro D, San-Segundo L, López-Corral L, Corchete LA, Gutiérrez NC (2015). In vivo murine model of acquired resistance in myeloma reveals differential mechanisms for lenalidomide and pomalidomide in combination with dexamethasone. Leukemia.

[13] International Myeloma Working Group (IMWG) Uniform Response Criteria for Multiple Myeloma [Internet]. Int. Myeloma Found. 2022. https://www.myeloma.org/resource-library/international-myeloma-working-group-imwg-uniform-response-criteria-multiple.

[14] Lahuerta JJ, Paiva B, Vidriales MB, Cordón L, Cedena MT, Puig N (2017). Depth of Response in Multiple Myeloma: A Pooled Analysis of Three PETHEMA/GEM Clinical Trials. J Clin Oncol..

[15] Kumar S, Jacobus SJ, Cohen AD, Weiss M, Callander NS, Singh AA (2020). Carfilzomib, lenalidomide, and dexamethasone (KRd) versus bortezomib, lenalidomide, and dexamethasone (VRD) for initial therapy of newly diagnosed multiple myeloma (NDMM): Results of ENDURANCE (E1A11) phase III trial. J Clin Oncol..

[16] Radhakrishnan VS, Tamboli N, Das S, Garg JK, Nag A, Bhave SJ (2021). Bortezomib-Pomalidomide-Dexamethasone (VPD) As Novel Induction Therapy in Newly-Diagnosed Multiple Myeloma: Early Safety Data from an Ongoing Single Arm Phase-II Investigator-Initiated Clinical Trial (PRIME Study). Blood.

[17] Zamagni E, Nanni C, Gay F, Dozza L, Rota Scalabrini D, Omedé P (2019). MRD Evaluation By PET/CT According to Deauville Criteria Combined with Multiparameter Flow Cytometry in Newly Diagnosed Transplant Eligible Multiple Myeloma (MM) Patients Enrolled in the Phase II Randomized Forte Trial. Blood.

[18] Cedena MT, Martin-Clavero E, Wong S, Shah N, Bahri N, Alonso R (2020). The clinical significance of stringent complete response in multiple myeloma is surpassed by minimal residual disease measurements. PloS ONE.

[19] Zamagni E, Nanni C, Gay F, Dozza L, Rota Scalabrini D, D’Agostino M (2020). Impact of Imaging FDG-PET/CT Minimal Residual Disease Assessment on Outcomes and Matching with Bone Marrow Techniques in Newly Diagnosed Transplant Eligible Multiple Myeloma (MM) Patients: Results of the Phase II Randomized Forte Trial. Blood.

[20] Culakova E, Thota R, Poniewierski MS, Kuderer NM, Wogu AF, Dale DC (2014). Patterns of chemotherapy-associated toxicity and supportive care in US oncology practice: a nationwide prospective cohort study. Cancer Med.

[21] Lonial S, Waller EK, Richardson PG, Jagannath S, Orlowski RZ, Giver CR (2005). Risk factors and kinetics of thrombocytopenia associated with bortezomib for relapsed, refractory multiple myeloma. Blood.

[22] Pancheri E, Guglielmi V, Wilczynski GM, Malatesta M, Tonin P, Tomelleri G (2020). Non-Hematologic Toxicity of Bortezomib in Multiple Myeloma: The Neuromuscular and Cardiovascular Adverse Effects. Cancers.

[23] Plummer C, Driessen C, Szabo Z, Mateos MV (2019). Management of cardiovascular risk in patients with multiple myeloma. Blood Cancer J.

[24] Yanamandra U, Malhotra P (2019). Stem Cell Transplantation in Multiple Myeloma: Very Much Alive and Kicking. Indian J Hematol Blood Transfus..

[25] Cowan AJ, Baldomero H, Atsuta Y, Mikhael J, Aljurf M, Seber A (2020). The Global State of Hematopoietic Cell Transplantation for Multiple Myeloma: An Analysis of the Worldwide Network of Blood and Marrow Transplantation (WBMT) Database and the Global Burden of Disease Study. Biol Blood Marrow Transplant.

[26] Kulkarni U, Devasia AJ, Korula A, Fouzia N, Nisham P, Samoon YJ (2018). Use of Non-Cryopreserved Peripheral Blood Stem Cells Is Associated with Adequate Engraftment in Patients with Multiple Myeloma Undergoing an Autologous Transplant. Biol Blood Marrow Transplant.

[27] Braunlin M, Belani R, Buchanan J, Wheeling T, Kim C (2021). Trends in the multiple myeloma treatment landscape and survival: a US analysis using 2011–2019 oncology clinic electronic health record data. Leuk Lymphoma.

